# Seed Train Optimization in Microcarrier-Based Cell Culture Post In Situ Cell Detachment through Scale-Down Hybrid Modeling

**DOI:** 10.3390/bioengineering11030268

**Published:** 2024-03-09

**Authors:** Atefeh Ebrahimian, Mona Schalk, Mark Dürkop, Michael Maurer, Rudolf Bliem, Harald Kühnel

**Affiliations:** 1 ACIB—Austrian Centre of Industrial Biotechnology, Krenngasse 37, 8010 Graz, Austria; 2 Department of Applied Life Science, Bioengineering, FH-Campus Wien, 1100 Vienna, Austria; 3 Department of Biotechnology, University of Natural Resources and Life Sciences, 1190 Vienna, Austria; 4 Novasign GmbH, 1020 Vienna, Austria

**Keywords:** seed training, MA 104 cells, microcarrier culture, in situ cell detachment, process optimization, hybrid modeling

## Abstract

Microcarrier-based cell culture is a commonly used method to facilitate the growth of anchorage-dependent cells like MA 104 for antigen manufacturing. However, conventionally, static cell culture is employed for cell propagation before seeding the production bioreactor with microcarriers (MCs). This study demonstrates the effective replacement of the conventional method by serial subculturing on MCs with in situ cell detachment under optimal conditions in closed culture units. This study proves that MA 104 can be subcultured at least five times on Cytodex 1 MC without the need for separating cells and MC after cell harvest. Process parameters impacting cell growth were studied post in situ cell detachment in a scaled-down model. Optimization, using augmented Design of Experiments (DoE) combined with hybrid modeling, facilitated rapid screening of the design space for critical process parameters (CPPs). Optimized conditions included an inoculation density of >16 cells/bead, 3.5–4.5 g/L of Cytodex 1, and a controlled agitation speed, starting at N_js_ (minimum agitation speed) for the first day with a maximum increase of 25% thereafter. With these design spaces for CPPs, a cell density of 2.6 ± 0.5 × 10^6^ cells/mL was achieved after five days. This refined bioprocess methodology offers a reliable and efficient approach for seed training in stirred tank reactors, which is particularly beneficial for viral vaccine production.

## 1. Introduction

The significance of vaccines in combating viral diseases, including the recent COVID-19 pandemic, is widely acknowledged. Vaccines stand as our most dependable defense against viral infections [1]. While recent advancements such as mRNA vaccines offer substantial advantages, drawbacks like rapid degradation after administration, reliance solely on the spike protein as an antigen, and stringent storage requirements persist [2,3]. 

Dead virus vaccines or attenuated vaccines address some of these challenges, providing stability, safety, and ease of production [4,5]. Within the production of inactivated or attenuated vaccines, a growing focus on cell culture-based processes, particularly using anchorage-dependent cell lines like Vero, MRC-5, or MA 104, necessitates the adoption of a bioprocess for adherent cultures [6]. 

Many manufacturers rely on static culture methods, such as roller bottles or multilayer culture systems, for cell propagation and final antigen manufacturing [7,8,9]. Rotarix^®^, Varivax^®^, Meruvax [9], Ervebo^®^ [10], PRRS [11], and rabies vaccines [12] for pigs are examples of the vaccines produced with static culture methods. However, these methods come with drawbacks, including restricted inoculation, limited monitoring of process parameters, substantial manufacturing space requirements, workforce limitations, and limited production scale. To overcome the challenges of static cell culture methods, microcarriers (MCs) have been introduced to provide a surface for adherent cell culture in a standard mixing vessel, as developed by van Wezel [13]. MCs offer a high surface-to-volume ratio, bringing culture into suspension, improving process consistency, and facilitating scale-up [7,14,15]. 

Currently, the accepted procedure for many virus production manufacturers involves starting the seed culture in static mode and then transferring the cells to the production bioreactor to continue growth on microcarriers [6]. Nevertheless, integrating microcarrier cultures into seed training is required if the desired inoculum exceeds the capacity achievable through static cell culture [16]. Considering microcarrier-based bioreactor cultures for seed propagation and employing the unit process scale-up methodology seem promising to overcome the complexities of static cell culture multi-unit scale-out [17]. 

In general, MC-based cell culture involves three main stages: cell attachment, proliferation, and detachment. The initial stages, that is, cell attachment to MCs and proliferation, which significantly impact the overall efficiency, have been extensively studied by different research groups [18,19,20,21]. This relatively simple approach, focusing mostly on optimizing different parameters with cells obtained from static cell culture, does not represent the real condition. Maximum achievable cells are different once the cells have undergone detachment from MC [22]. 

For cell detachment on MC, a combination of enzymatical and mechanical methods has been used [23,24]. After cell detachment from MC, two options exist for transferring to the larger scale: first, transferring only single cells after the MCs have been settled or separated by mesh [14,24,25]; second, transferring previously used MCs (spent MCs) simultaneously with detached cells to the next bioreactor (bead to bead transfer, b2b) [14,17,26,27]. The first option typically results in cell loss at harvesting [14] due to the lack of a deep tube in standard stirred bioreactors above settled MCs, when MCs are separated from cells by settling. Additionally, increased reactor height prolongs settling time on a larger scale, which results in increased cell loss. Moreover, using intermediate equipment like a mesh screen to separate MCs from cells, results in a more complicated process [22,25]. The second option of simultaneous transfer renders higher cell recovery and has been proven to work for scales of up to 1200 L on Cytodex 3, albeit not optimized [26]. This second option is generally more promising, yet its effectiveness needs validation for each cell line. Although certain studies confirm b2b transfer for the subjected cell line, no study has investigated the impact of CPPs after cells undergo in situ cell detachment from MC. 

This study aims to propose a reproducible and scalable seed training method for MA 104 cell line on Cytodex 1 as MC. MA 104 is an epithelial cell line from the kidney of an African green monkey, which is commonly used for simian rotavirus production as it is highly susceptible to this virus [28,29,30,31]. In the first part, the consecutive in situ cell detachment–attachment of the cells of MA 104 cell line is demonstrated. 

In the second part, a three-dimensional experimental design was created based on the following three CPPs: seeding density (cells/bead ratio), MC concentration, and agitation speed. Cellular growth was described with a hybrid modeling approach to identify optimal growth conditions in a scale-down strategy. Utilizing a fully operational bioreactor with a volume of 700 mL, we simulated conditions representative of the entire upscaling process. The study assesses process limits and provides recommendations for achieving optimal outcomes across all scales.

## 2. Materials and Methods

### 2.1. Cell Line and Culture Medium

The cell line MA 104, that is, African green monkey kidney cells, was used. The cells were stored in a nitrogen tank before thawing and expansion using Glasgow medium (ThermoFisher Scientific, Inchinnan, Scotland) containing 1% of Penicillin-Streptomycin solution (5000 U/mL) (Gibco, New York, NY, USA) and supplemented with 10% FBS (SAFC Bioscience Inc., Brighton, VIC, Australia) and 2% (*w*/*v*) of peptone (NEOGEN, Lansing, MI, USA).

### 2.2. Subculture in T-Flask

Cells in tissue culture flasks (175 cm^2^, 225 cm^2^) were routinely subcultured to 0.15–2 × 10^4^ cells/cm^2^ every 2–3 days when confluency of more than 85% was reached. Cells were grown at 37 °C with 5% CO_2_ in a humidified incubator. Cell detachment procedures were performed using 0.02 mL of 0.25% Trypsin-EDTA (ThermoFisher Scientific, Waltham, MA, USA) per cm^2^ growth area, following a one-time PBS wash (DPBS solution, pH = 7.4, VWR, USA, Pennsylvania). 

### 2.3. Subculture on MCs by Consecutive Cell Detachment–Attachment without Cell and MC Separation

Spinner cultures were carried out in Wheaton and VWR vessels (200 mL working volume) at 37 °C in an incubator on a spinner platform (Scientific Industries. Watertown, MA, USA). Mixing was set to 60 rpm, in a continuous way, starting with inoculation and MC colonization. The spinners were inoculated with a cell density of 0.2 × 10^6^ cells/mL, and a Cytodex 1 (Cytiva, Marlborough, MA, USA) concentration of 3 g/L was used for each spinner culture. The MC stock solution was prepared according to the manufacturer’s instructions. The seed for the first spinner was obtained from cells harvested from T-flasks. During the culture, 50% of the used medium was replaced by fresh, rich medium (10% FBS) on the 2nd and 4th days of culture. Medium replacement was carried out by stopping the impeller and letting the MCs settle. The sedimentation velocity of Cytodex 1 in culture medium was measured in-house and assessed as 70–75 cm/h.

**Figure 1:**
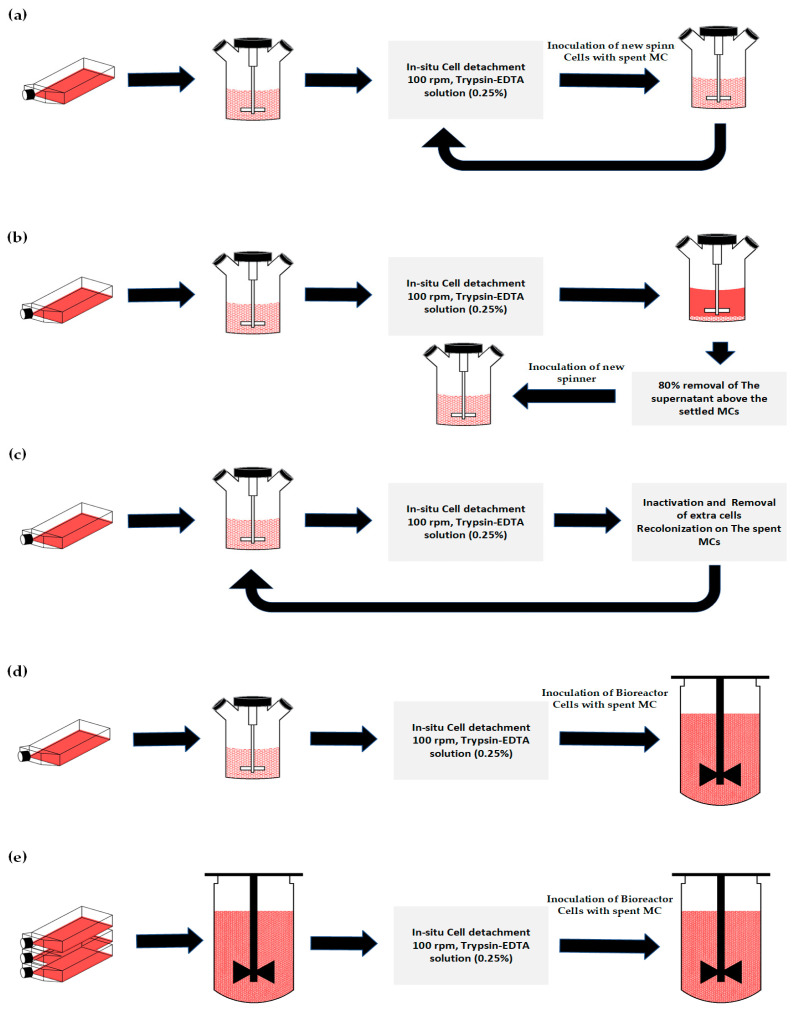
Method of the establishment of b2b transfer for MA 104 cell line: (**a**) method of subculturing of cells on MCs via consecutive cell detachment–attachment without separating cells and MCs, (**b**) method of subculturing by cells and MCs separation after in situ cell detachment, (**c**) method of recolonization capability of spent MC in a consecutive cell detachment–attachment, (**d**) method of seeding from spinner to the bioreactor for the DoE study, and (**e**) method of subculturing in two consecutive bioreactors.

After reaching the cell confluency on MC, in situ cell detachment was performed in the spinner (Figure 1a). For the cell detachment from MC, a Trypsin-EDTA 0.25% solution with a ratio of 90 mL/g of MC was used. For in situ cell detachment, the following steps were conducted: (1) stop the agitation and allow MC to settle, (2) remove 80% of the used medium, (3) wash MC twice with PBS, pH 7.4 (without Ca^2+^ and Mg^2+^) with the ratio of 40% of the initial volume (iv), and (4) add Trypsin solution and mix at 100 rpm for 25–30 min. The complete detachment of cells from MC was monitored under the microscope by sampling throughout the cell detachment. After achieving complete cell detachment, the cells and the spent MC were transferred with a split ratio of 1:5 to a previously prepared spinner, as outlined. The process of in situ cell detachment and attachment was repeated 5 times.

### 2.4. Subculture on MCs by Cell and MC Separation after In Situ Cell Detachment

After performing in situ cell detachment in spinner culture, as detailed in Section 2.3, the Trypsin solution was neutralized by adding Glasgow medium containing 10% FBS to achieve a final culture volume of 200 mL. Subsequently, agitation was stopped, allowing the MCs to settle. Then, 80% of the culture volume above the settled MCs was harvested and transferred to a sterile glass bottle. The rationale for removing only 80% was to align with a scaling-up approach. In larger-scale single-use bioreactors, such as Eppendorf single-use bioreactors, the deep tube accommodates only 80% of the working volume, making it difficult to harvest more cells.

The viable cell density of the harvested cells was determined using the method outlined in Section 2.9, and the necessary volume of the cell suspension was then transferred to a previously prepared spinner culture, as described in Section 2.3, to achieve an initial cell density of 200,000 cells/mL. Subsequent cell growth was monitored throughout the cell culture (Figure 1b).

### 2.5. Repetitive Recolonization of the Spent MC

Starting from a spinner culture under the conditions outlined in Section 2.3, in situ cell detachment was performed upon reaching confluency on MC. The concentration of single cells was determined by Trypan blue method (Section 2.9). The Trypsin solution was inactivated by adding culture medium (containing 10% FBS) to achieve a total volume of 200 mL. Agitation was stopped to allow the MCs to settle, and the portion of the cell suspension necessary to attain a cell concentration of 0.2 × 10^6^ cells/mL in the final culture volume of 200 mL was determined. Following the removal of excess cells, the culture medium was added to restore the volume of 200 mL (Figure 1c). 

### 2.6. MC Culture in the Bioreactor for the DoE Study 

Bioreactor culture was carried out in the DASGIP system (Eppendorf, DASGIP^®^ Bioblock parallel system, Germany—700 mL), which was set up, calibrated, and controlled using the DASGIP DASbox control unit (Eppendorf, Hamburg, Germany). All bioreactors were equipped with a 30° angled 3-blade impeller. Glasgow medium, including 0.1% of Pluronic F-68 (ThermoFisher Scientific, Waltham, MA, USA), was used as culture medium.

The bioreactors were filled with the culture medium and MC, mixed, and aerated for MC conditioning and sterility check one day before inoculation. The required cells were transferred from the spinner culture after in situ cell detachment, conforming to the spinner conditions outlined in Section 2.3 (Figure 1d).

For the first 24 h post-inoculation, agitation was set at 80 rpm as the minimum agitation speed (N_js_) required to keep the MC from settling on the reactor bottom. This N_js_ value was experimentally determined for this scale, as explained in Section 2.8. For the DoE study, the MC concentration, the required seeding density (cells/bead ratio), and the agitation speed were varied based on predefined experimental runs in Section 2.10.2. During the entire culture period, the minimum dissolved oxygen (DO) was set at 50% saturated air and controlled by the addition of air enriched with a maximum of 40% oxygen through sparging. Within the first 24 h post-inoculation, sufficient aeration could be achieved through headspace aeration with 0.05 VVM. The pH was controlled between 7.2 and 7.4 by the addition of NaHCO_3_ (7.5% solution) and CO_2_ gas (with a maximum concentration of 10%). 

Furthermore, 50% of the present depleted medium was replaced by fresh, rich medium (10% FBS) on the 2nd and 4th days of culture. Following the same procedure as in the spinner culture, the MCs were left to settle, and then 50% of the spent medium was replaced by the same volume of fresh and pre-warmed culture medium, after which the agitation and aeration were turned on again. 

### 2.7. Subculturing in Two Consecutive Bioreactors 

Bioreactors were set up following the procedure described in Section 2.6. In the first bioreactor, the seeding density was 15.5 cells/bead, using cells harvested from flask cultures. MC concentration and agitation speed were set at 3 g/L and 100 rpm, respectively. Once cell confluency on the microcarriers had been achieved, in situ cell detachment was conducted within the bioreactor, following the procedure described in Section 2.3. Liquid handling and transfers were controlled using pumps and weight balances. Upon achieving total cell detachment (after 10–15 min), the required volume of cell suspension for transfer to the next bioreactor was calculated to achieve an initial seeding density of 20 cells/bead.

The cell suspension, including the spent MC, was conveyed through a connected tube using a calibrated pump (see Figure 1e). After cell transfer, the bioreactor volume was adjusted to the maximum working volume of 700 mL. Agitation speed was started at 80 rpm for the first day, increased to 90 rpm for the 2nd and 3rd days, and set to 100 rpm thereafter. Cell growth was monitored throughout the culture.

### 2.8. Minimum Agitation Speed Determination

The minimum agitation speed (N_js_—js stands for just suspended) for MC suspension in a 1 L DASGIP bioreactor was experimentally determined. Agitation speed at N_js_ guarantees MC suspension in the bioreactor culture [23,32]. Samples were taken from a suspension of a known MC concentration of 3 g/L in Glasgow culture medium at different agitation speeds. At each agitation speed, samples with a volume of 10 mL were taken from the top and bottom of the vessel, respectively. MCs in the 10 samples were collected by passing through a pre-weighed strainer, 70 μm (Greiner Bio-One, Kremsmünster, Austria). The actual MC concentration was determined through the measurement of mass after drying at 55 °C for at least 3 days. The optimum agitation speed, which resulted in the lowest concentration gradient (i.e., difference in MC concentration at the top and bottom of the vessel) and prevented MC settling, was found to be 80 rpm.

### 2.9. Sampling and Analytical Methods

For the analysis, 4 mL of samples were taken from both spinner and bioreactor cultures daily. Total cell concentration was measured using 0.1% crystal violet solution (ESCO Bioengineering, Taiwan), using the method described by Souza et al. [21]. 

Viable single cells obtained from in situ detachment or T-flask were counted by the Trypan blue exclusion method with Countess (ThermoFisher Scientific, Waltham, MA, USA). The recovery of in situ cell detachment was calculated based on the total viable single cells after complete cell detachment over the total cell concentration measured by the crystal violet assay.

Glucose and lactate were analyzed via high-performance liquid chromatography (HPLC, ThermoFisher, Massachusetts, USA) employing an anion exchange column (Aminex HPC-87H, Bio-Rad, Hercules, CA, USA). The method was adapted from the column manufacturer’s instructions, conducting an isocratic separation using a running buffer of 5 mM sulfuric acid at a flow rate of 0.35 mL/min. In this setup, UV signal and refractive index (RI) were utilized to, respectively, detect lactate and glucose. Standard calibration solutions were prepared using HPLC-grade glucose and lactate within an appropriate concentration range.

### 2.10. Hybrid Modeling of Cellular Growth on MC after In Situ Cell Detachment

#### 2.10.1. Parameter Selection for the DoE Study

The potential CPPs for MC-based cell culture were identified through risk assessment and preliminary experiments. Various parameters, including pH, glucose content, aeration strategy, MC concentration, initial cell density, and agitation speed, were studied. Based on the conducted experiments, three CPPs and their experimental ranges were determined, namely MC concentration, seeding density, and agitation speed. The levels for the three factors are 3, 4, and 5 g/L; 4, 12, and 20 cells/bead; and 70, 100, and 130 rpm, respectively. Within these three parameters, both MC concentration and seeding density (cells/bead) exhibit scale independence, whereas agitation speed changes on larger scales. The methodology to identify the experimental range of agitation speed for a 1 L bioreactor was based on the mean Kolmogorov length scale (λ) and N_js_, which are scalable parameters. 

Typically, analyses of mechanical stresses on biological entities are predicated on the Kolmogorov theory of isotropic turbulence [33]. The existing literature suggests that λ should be sufficiently large and exceed two-thirds of the mean MC size [34] to minimize fluid shear-induced cellular damage on MCs [14,35,36,37]. 

The microscale of λ can be computed using the following formula:

(1) $$P=N_{P}\times \rho \times N^{3}\times D_{i}^{5}$$

(2) $$\bar{\varepsilon_{T}}=\frac{P}{\rho \times V}$$

(3) $$\lambda ={\left(\frac{\upsilon^{3}}{\bar{\varepsilon }}\right)}^{0.25}$$

$\bar{\varepsilon_{T}}$ = mean specific energy dissipation rate (m^2^/s^3^)
ν = kinematic viscosity (cm^2^/s)
P = power consumption (kg.m^2^/s^3^)
ρ = density (g/cm^3^)
V = working volume of the reactor (cm^3^)
N_p_ = power number of the bioreactor
N = agitation speed (1/s)
D_i_ = impeller diameter.

This formula (Equation (1)) requires turbulent flow in the cultivation, that is, a high Reynolds number of over 10^4^. Although the flow in the experimental range for agitation speed is in the transition phase and only moderately turbulent, according to Table 1, it is justifiable to use the turbulent flow approach for calculation based on the explanation by Hewitt et al. [35]. 

The average size of MCs (d50) in Cytodex 1 was determined in-house by examining over 1000 individual MCs through an optical microscope. The analysis involved the curve fitting of a normal distribution to the data using GraphPad Prism 10.0 (Supplementary Materials, Figure S1), resulting in a confirmed average size of 181.0 µm. Therefore, even at the highest agitation speed of 130 rpm, λ does not exceed 2/3 of d50, which stands at 120 µm.

For the lower limit of agitation speed, the N_js_ value was considered. However, given the experimental nature of this parameter (outlined in Section 2.6), a slightly lower initial agitation speed of 70 rpm was adopted to initiate the DoE study, aiming to include a broader scope and explore potential correlations with the other two parameters of MC concentration and seeding density.

**Table 1:** Operational conditions at the experimental range for agitation speed.

Agitation Speed (rpm)	Tip Speed (m/s)	Re	$\bar{\varepsilon }$ (m^2^/s^3^)	λ (μm)
70	0.183	2607	0.000106	190.50
100	0.262	3724	0.0031	145.79
130	0.340	4841	0.0068	119.75

Bioreactor type: DASGIP DASBOX Eppendorf Reactor, N_p_ = 1.5 (for tip speed less than 0.4) [38]; working volume (WV): 700 mL; impeller type: 3-blade segment, 30° angled; D_i_ = 5 cm; medium: Glasgow medium contains 10% FBS, ν = 0.95 cP; ρ = 1.01 g/cm^3^.

#### 2.10.2. DoE Study

Following an assessment of the CPPs and their levels, a three-dimensional full factorial design 3^3^ was considered. Due to constraints on time and resources, only 21 runs were performed out of the full design. Therefore, a stepwise augmented design was considered, and up to 21 bioreactor culture runs in the DASGIP system were performed. The fractional approach was explored, starting with the extreme scenarios. For example, out of the initial four cultures, two cultures were conducted at the lowest MC concentration (3 g/L), while seeding density and agitation speed covering both the lowest and highest ranges. The remaining two cultures were executed at the highest MC concentration, encompassing both the lowest and highest agitation speed and seeding density. The list of all conducted cultivation experiments for the designed experiments is provided in the Supplementary Materials (Table S1). All cultures in the DoE study were performed based on the details explained in Section 2.5. The ranges of the parameters of seeding density and MC concentration deviate from the defined experimental range due to the transfer of spent MCs from the spinner culture (refer to Section 2.6). 

#### 2.10.3. Hybrid Modeling 

For process modeling, the online datafiles for each cultivation were exported from DASWARE (Eppendorf, Juelich, Germany) and converted into a single data file containing all time-aligned online and offline data. This dataset is also available in the Supplementary Materials (File S1.xlsx). The dataset was then imported into the commercially available version of the Hybrid Modeling Toolbox (Novasign GmbH, Vienna, Austria). 

The tool automatically scales all input variables (MC concentration, seeding density, and agitation speed) using the z-score. The non-parametric part of the hybrid model utilized an artificial neural network (ANN) with one hidden layer and 3 nodes, applying a tangential hyperbolic transfer function for the hidden layer and a linear transfer function for the output layer. The purpose of the ANN was to estimate the current growth rate at each time interval as a function of all available inputs, namely the MC concentration, seeding density, and agitation speed. The resulting estimated growth rate was fed into the mechanistic part of the hybrid model (Equation (4)).

(4) $$\frac{dX}{dt}=\mu \times X$$

The cellular growth rate µ is thereby a function of the current values of microcarrier concentration, agitation speed, and the initial seeding density, as well as the biomass present at the previous prediction (X_t−1_). The datasets were split into different distributions (boots) for validation purposes. In each boot, 14 of the runs were used for training, and 7 full datasets were used for validation. Levenberg-Marquardt regularization was applied to minimize the error between the datasets and the model, similar to [39,40]. Overall, 20 different data distributions (boots) were generated. Thereby, the algorithm is not more or less prone to data overfitting. Finally, the best models were selected from the 5 different boots and averaged according to a previously described methodology [41]. After the finalization of the model structure, an offline digital twin of the process was created to simulate all potential process scenarios and identify ideal process conditions. Based on the built hybrid model, a digital twin of the process was created in silico, consisting of 2808 in-silico runs spanning the entire experiment range. This twin can be retrained on every new run, allowing advanced process understanding, and is directly used for soft sensing when fetching CPPs from the operation process control (OPC) values of the DASGIP reactor. 

## 3. Results

### 3.1. Subculture on MCs by Consecutive Cell Detachment–Attachment without Cell and MC Separation

**Figure 2:**
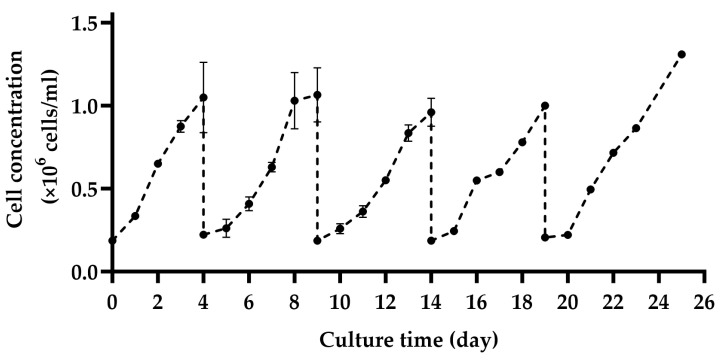
Reproducibility assessment of subculturing by several cycles of cell detachment and resettling of MA 104 cells on Cytodex 1 without separating the cells from the spent MCs.

Figure 2 illustrates the reproducibility of five instances of in situ cell detachment and colonization of MA 104 cell line on Cytodex 1, following the method outlined in Section 2.3. As shown, after each in situ cell detachment and subsequent colonization of the MCs, the cell concentration reached a peak at an average of 1.1 × 10^6^ cells/mL within 4–5 days of culture. In Figure 2, the cells were cultivated in shake flasks for the initial four days. However, the following cell growth represents cells harvested through in situ cell detachment by Trypsin EDTA solution. Hence, the specific cell growth for the initial four days on MC is higher. Yet, there were no notable differences in the final cell numbers achieved after 4–5 days. The result shows that MA 104 cells can undergo multiple cycles of detachment and resettling on Cytodex 1 without changing their growth behavior. Images in the supplementary materials (Figure S2) show optical and fluorescent microscopic observation of the cells on MCs as exemplary of MA104 cell growth on MCs after undergoing multiple in situ cell detachments. 

As outlined in Section 2.3, a continuous agitation speed has been used since the inception of inoculation. The graph presented in the supplementary materials (Figure S3) illustrates that there is no difference in the colonization phase (the first 6 h of culture) between intermittent and continuous stirring. 

### 3.2. Subculture on MCs by Cell and MC Separation after In Situ Cell Detachment 

**Figure 3:**
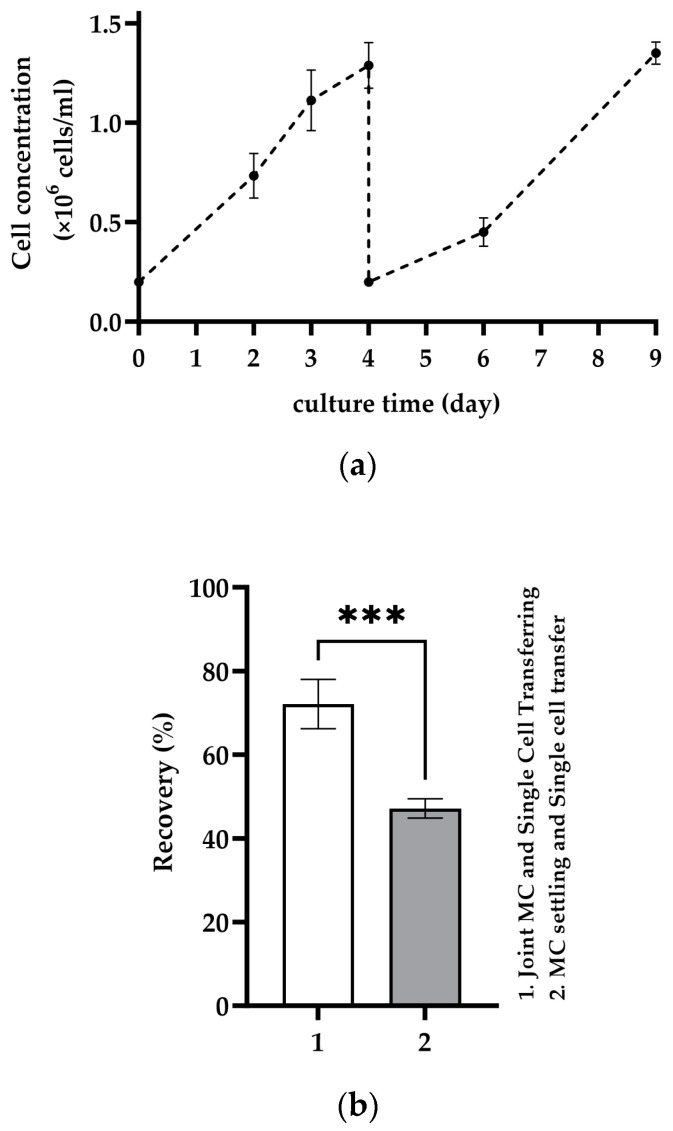
(**a**) Cell growth trend upon exclusive transfer of single cells after in situ cell detachment and (**b**) total cell recovery from two methods of jointly transferring cells and MC or only transferring the single cells after MC settling (*p* ≤ 0.001: ***).

Figure 3a outlines the growth trend of cells after transferring only the individual cells following in situ cell detachment. This growth was equal to the growth observed when cells were transferred jointly with the spent settled MC (Figure 2). Nevertheless, the overall cell recovery was less compared to the simultaneous transfer of cells and MCs when only single cells were transferred. In Figure 3b, the difference in cell recovery between both methods is illustrated. The average cell recovery during in situ cell detachment exceeded 70%. In contrast, when considering the separation of MCs and cells—giving cells time to settle, and only being able to harvest 80% of the medium above them—the maximum cell recovery was less than 50%.

Throughout the cell transfer process after enzymatic cell detachment, including both exclusive transfer of detached cells as well as joint transfer of cells and spent MCs, the cells were able to re-attach to Cytodex 1. In both transfer methods, only a negligible occurrence of cell and MC aggregation was observed. 

### 3.3. Repetitive Recolonization of the Spent MC

**Figure 4:**
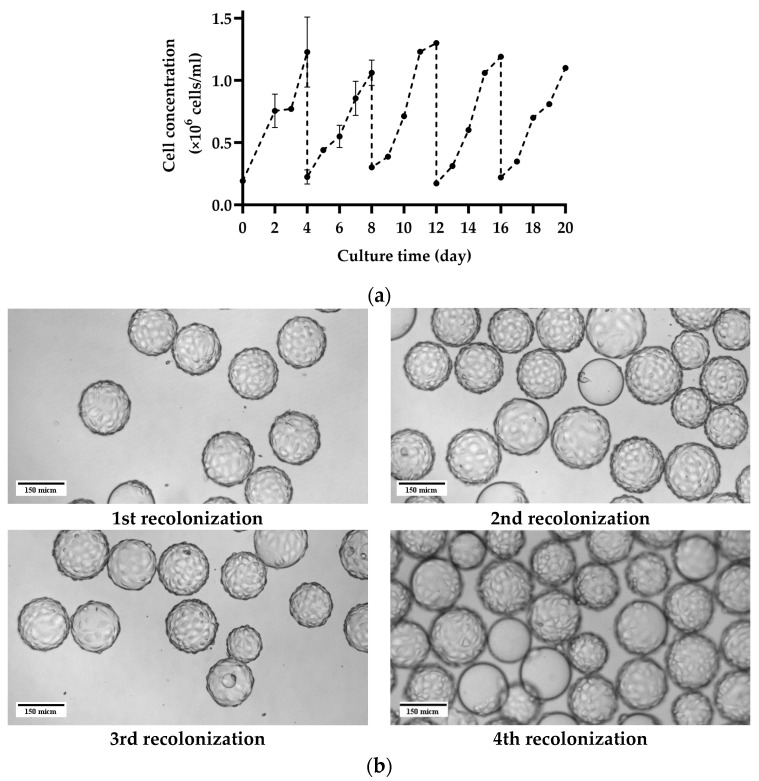
(**a**) Reproducibility of cell detachment on the MC and recolonization of MA 104 cells on the same Cytodex 1 and (**b**) cell growth on Cytodex 1 after 4 days of culture in each recolonization on the same Cytodex 1.

Figure 4a demonstrates the Cytodex 1 microcarrier’s capability to support repeated recolonization by MA 104 cells. Results show that after each recolonization, an average cell density of over 1 × 10^6^ cells/mL was achieved within 4 days of cultivation, with consistent cell growth across multiple in situ cell detachment cycles. As illustrated in the graph, the initial seeding density at each recolonization was between 150,000 and 300,000 cells/mL. Achieving reproducible seeding density proved challenging, resulting from the need to remove excess cells during MC settling after complete cell detachment, thereby increasing cell loss due to cells settling with the MC. Images in Figure 4b show optical microscopic observations of MA 104 cells on Cytodex 1 on the 4th day of culture in each recolonization. The observations confirm the monolayer growth of MA 104 cells on the same spent Cytodex 1 after each recolonization cycle. Notably, observed cell aggregation on Cytodex 1 was minimal, occurring on less than 6% of the total MCs.

### 3.4. Hybrid Modeling of Cellular Growth on MC after In Situ Cell Detachment 

**Figure 5:**
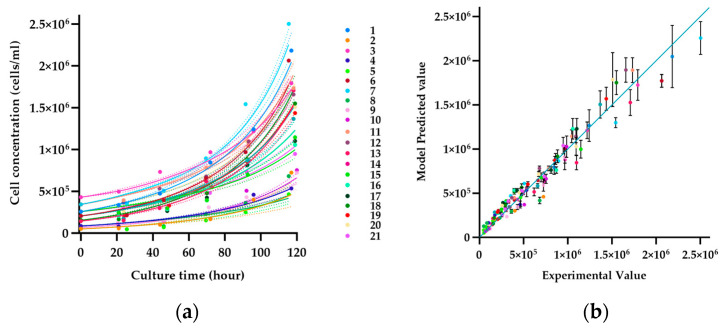
(**a**) Comparative evaluation of the predictive quality of the developed hybrid model with experimental data, colored continuous lines (predicted values), and colored squares (experimental result). (**b**) Accuracy of concatenated predictions over experimental value. Each color represents one experimental run in DoE.

Figure 5a,b indicate the predictive performance of the hybrid model developed according to Section 2.10.3. It predicts the total cell concentration during 5 days of culture based on the selected process parameters in different runs (see Supplementary materials, File S1 and Table S1). The responses of cell growth to the different process parameters (colored squares) as well as the modeling accuracy are depicted (difference between solid lines and colored squares). It is visible that the processes resulting from the chosen input parameters differ significantly, yielding between 0.5 and 2.5 × 10^6^ cells/mL at the end of the culture. The hybrid model could describe the behavior accurately over the full-time course with a root mean square error (RMSE) of 92,705.18 cells/mL. Thus, the model could illustrate in detail the propagation of cells over the full process duration, taking into account the three different input parameters.

**Figure 6:**
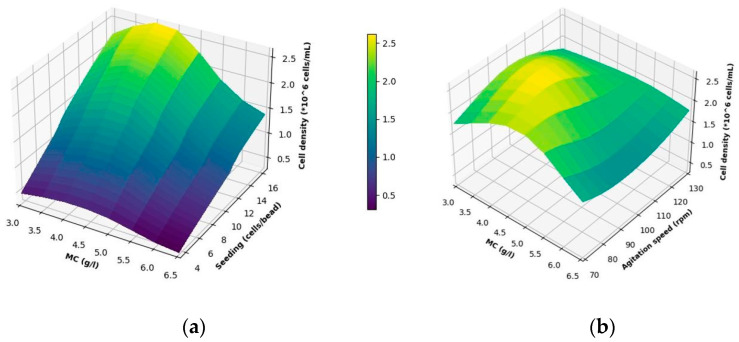
Influence of MC concentration, seeding density, and impeller speed on total cell concentration (cells/mL) after 120 h of cultivation. (**a**) Correlation of seeding density and MC concentration at 95 rpm. (**b**) Correlation of agitation speed and bead concentration at an actual seeding density of 16.5 cells/bead. Each grid point represents an in-silico experiment. Therefore, the final surface is a response from all experiments with linear interpolation between each condition. (*10^6 is equivalent to × 10^6^).

Figure 6a,b show the surface plots of in-silico simulation, which is built on selected models described in Section 2.10.3. These depict the predicted achievable total cell concentration (cells/mL) in the experiment as a function of the inputs. Figure 6a shows the surface plot of endpoint values of the final achievable cell concentration (cells/mL) as a function of MC concentration (g/L) and seeding density (cells/bead). Figure 6b presents a similar plot with a focus on MC concentration (g/L) and agitation speed (rpm). These figures highlight the importance of each CPP on the output and guide the selection of design space to achieve maximum cell concentration.

As can be observed from Figure 6a,b, the lower limit of response is 0.31 ± 0.85 × 10^6^ cells/mL at a seeding density of 3.5 cells/bead, an MC concentration of 6.5 g/L, and an agitation speed of 95 rpm, while the upper limit is 2.6 ± 0.5 × 10^6^ cells/mL at a seeding density of 16.5 cells/bead, a MC concentration of 4 g/L, and an agitation speed of 95 rpm. 

For the parameter of seeding density (Figure 6a), a maximum cell concentration of 2.6 × 10^6^ cells/mL was predicted at the highest seeding density of 16.7 cells/bead at MC concentration and agitation of 4 g/L and 95 rpm, respectively. Based on the outcomes of this DoE study, described by the digital twin (Figure 6a,b), going further to higher cells/bead ratios should be considered to aim at achieving an even higher cell concentration at the end.

As depicted in Figure 6a, the impact of the MC concentration was not strong at unfavorable low seeding densities but became more pronounced at the highest seeding density. Here, a peak was reached at an MC concentration of 4.0 g/L, while at both higher and lower MC concentrations, the achievable cell numbers were reduced. However, decreasing or increasing MC concentration to 3.5 g/L and 4.5 g/L, respectively, did not yield a notable difference in the achievable cell density (Supplementary Figure S4a).

Similar results were obtained in Figure 6b for the impeller speed. It showed a peak at 95 rpm at 4 g/L of MC concentration and the highest actual seeding density of 16.5 cells/bead. In this figure, a decrease can be observed towards both extreme values. However, being in the range of 90–100 rpm while keeping the other two parameters at optimum conditions did not significantly change the achievable cell density (Supplementary materials, Figure S4b). 

The simulation predictions suggest a favorable design space characterized by an agitation speed ranging from 90 to 100 rpm, a microcarrier (MC) concentration between 3.5 and 4.5 g/L, and a seeding density exceeding 16.5 cells/bead. This combination ensures achieving a high cell density exceeding 2.0 × 10^6^ cells/mL. 

### 3.5. Validation of Predicted Culture Conditions in Two Consecutive MC Cultures in a Bioreactor 

**Figure 7:**
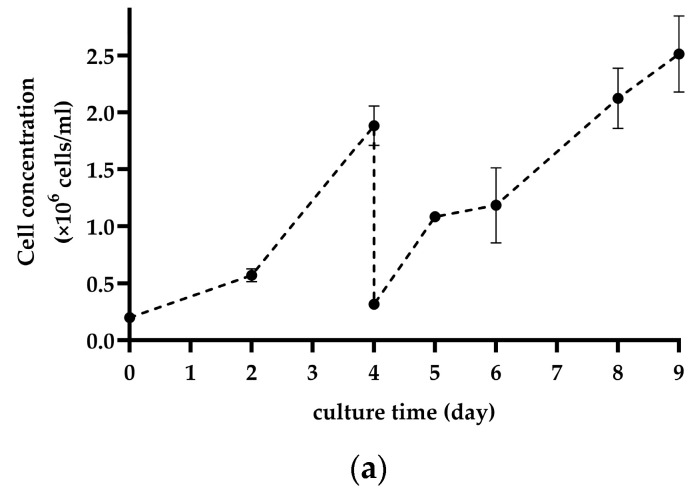

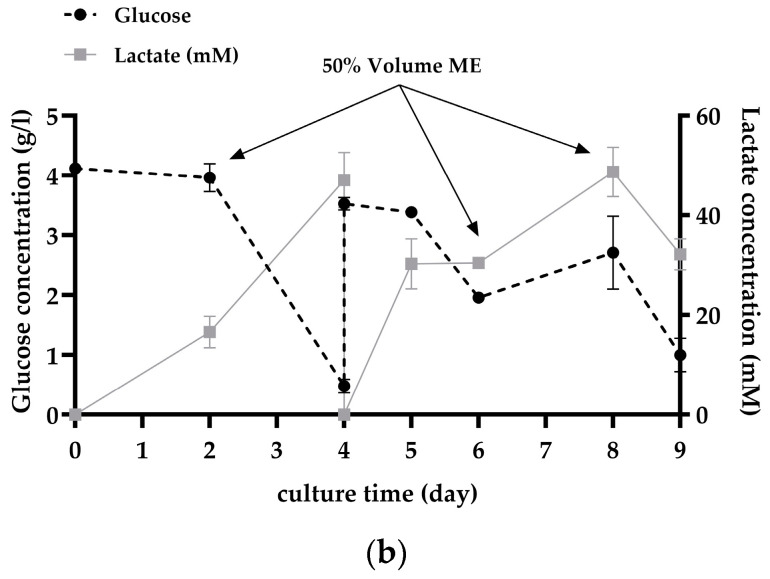
(**a**) Cell growth on MC in optimum condition after in situ cell detachment and (**b**) glucose and lactate concentrations over the culture time in the bioreactor (ME: medium exchange). The points are the mean values of two bioreactor cultures.

Figure 7a,b illustrates the cell growth, glucose, and lactate concentration in the optimized condition following in situ cell detachment in the bioreactor, as outlined in Section 2.7. The initial four days of culture were conducted in the first bioreactor, seeded with cells that were harvested from the flask. The subsequent 5 days of culture were followed by in situ cell detachment. The CPPs were in the design space determined by hybrid models: MC concentration of 3.6 g/L, seeding density of 16.86 cells/bead, and agitation speed of 90–100 rpm. After in situ cell detachment, the cell concentration reached 2.5 × 10^6^ cells/mL, which is in perfect agreement with the predictions of the model for these process conditions. In this culture, as described in the method, two rounds of medium exchange (ME) were conducted on the 2nd and 4th days of culture with fresh medium containing 10% FBS. However, the second medium exchange can be performed with a medium containing no FBS without any impact on the cell growth trend (Supplementary materials, Figure S5). 

## 4. Discussion

### 4.1. Subculture on MCs by Consecutive Cell Detachment–Attachment without Cell and MC Separation

Determining the most effective method to generate the large cell numbers required for vaccine manufacturing is critical for scaling up the process. In this research study, we demonstrated that MA 104 cells can undergo multiple cycles of in situ cell detachments and recolonize both fresh and spent Cytodex 1. Consequently, the consecutive cell detachment–attachment method proves to be a viable approach for seed training and cell propagation to achieve enough cells for antigen manufacturing. 

Cell lines may exhibit varying behaviors in response to different seed training methods. For instance, studies by Yang et al., 2019 [14], and Sousa et al., 2019 [24], have shown that Vero cells, which have a similar origin to the studied MA 104 cell line, can regrow on MCs (Cytodex 1) after undergoing enzymatic cell detachment at least three times without observation of any aggregation of MCs or cells. Wu et al. (2015) also described a methodology involving two rounds of in situ cell detachment with Vero cells, conducted across a 3 L spinner and a 50 L bioreactor. They observed no evidence of cell aggregation or significant morphological changes upon scaling up to a final volume of 200 L [17]. Conversely, Yang et al. 2019 observed that HEK cells and Cytodex 1 tend to aggregate during a joint transfer of cells and MCs to the next cultivation unit, which was a wave bioreactor in this case [14]. 

Using the method of cell detachment from MCs is crucial for optimal seed training. Some studies suggest the possibility of cell migration from confluent MCs to newly added bare MCs through bridging mode as a method of seed training and scaling up [42,43,44,45]. This non-enzymatic bridging mode prevents any potential physiological damage to the cells [45]. However, the reported recovery of this method is lower compared to the detachment and re-attachment approaches, 59 ± 19% compared to 85 ± 4 [24]. Applying this method yields different growth behaviors between the new MCs and the already confluent ones. New MCs take more time to reach confluence, while the rest are already confluent with cells in the exponential growth phase or already in the stagnation phase [14]. Moreover, optimization of parameters like time, number of new MCs, and intermittent agitation aggravates the complexity of the process. 

In our study, continuous agitation was applied throughout all cell colonization steps on MCs. We did not use intermittent agitation to establish consecutive cell detachment–attachment, as it does entail difficulties in not only the operation but also hardware development. Mainly, an additional control system is required to promote intermittent stirring [46]. Intermittent stirring is frequently recommended to increase contact time at the time of cell colonization on MCs [24,47,48,49]. For instance, Lue et al. (2021) carried out experiments to assess the most effective stirring method in spinner flasks [49]. They discovered that extending the duration of intermittent stirring led to better cell attachment, with the optimal condition identified as 3 min of stirring, followed by 42 min of standing over 5 h. However, it is worth noting that this observation was made only in small-scale experiments and has yet to be confirmed in larger scale settings.

In addition to the limited scalability associated with intermittent stirring, this method poses the risk of oxygen limitation [7] and aggregation of cells and MC [43] due to insufficient mixing. Implementing intermittent stirring at scales larger than 250 L requires long time intervals for the settling of MCs and cells due to the large-scale reactor height. Extended periods of no agitation increase the risk of oxygen limitation. Furthermore, the non-uniform size profile of Cytodex 1 leads to an uneven cell distribution since heavier MCs settle faster, resulting in smaller MCs settling in a top layer that is more accessible to the cells. Therefore, our study excluded intermittent stirring or static incubation during the cell colonization step. 

### 4.2. Subculture on MCs by Cell and MC Separation after In Situ Cell Detachment

The practicality of exclusively transferring detached cells, excluding spent MCs, is limited owing to a substantial number of cells co-settling with the MCs during the necessary agitation stop, leading to decreased whole-cell recovery. Yang et al. (2019) suggested washing MCs two or three times with culture medium after settling to enhance cell recovery [14]. Nevertheless, additional steps pose risks of contamination and batch-to-batch variation. Moreover, allowing MCs to settle in larger-scale operations requires more time, and repetitive stops increase the risk of cell damage due to oxygen limitation. 

Additionally, in the context of commercial single-use bioreactors for scaling up, the longest deep tube only allows for the removal of 80% of the working volume, that is, in the best-case scenario, a maximum of 80% of detached cells can be collected, leading to an overall yield decrease. Designing a custom single-use bioreactor with a deep tube precisely above the level of settled MCs would significantly increase costs. In contrast, for the joint transfer of cells and MCs, a standard harvest tube touching the bottom of the bioreactor can be utilized.

Furthermore, incorporating intermediate equipment such as Harvestainer^TM^ [50] or other mesh screens [25] to separate microcarriers from cells adds an additional transfer step at each scale, resulting in a more complex and costly process.

### 4.3. Repetitive Recolonization of Spent MC 

In this investigation, the ability of MA 104 cells to recolonize only the spent MC was observed. No additional step was introduced to cleanse the MCs after cell detachment; instead, the spent MCs were directly re-seeded with cells. To our knowledge, this capacity for recolonization on the spent MCs has not been demonstrated in prior studies. Wang (1999) demonstrated the reuse of MCs (Cytodex-3), but only after washing and removing cell debris via incubation with PBS containing 0.01% EDTA at a pH of 6.0 and a temperature of 4 °C for 24 h [44]. Meanwhile, our results indicate that cells can actively and directly recolonize even on the spent Cytodex 1, providing a promising approach for improved scalability and efficiency of MC-based cell culture systems. 

### 4.4. DoE Study and Hybrid Process Modeling Outcome 

The conducted DoE study and the subsequent process hybrid model established based on the data determined the impact of each CPP on the achievable total cell concentration at any time during the process. The concluded design space conditions from the hybrid model (seeding density of >16 cells/bead, Cytodex 1 concentration of 3.5–4.5 g/L, and agitation speed of 90–100 rpm = 1.125–1.25 × N_js_) are in good alignment with the process knowledge, and an explainable and meaningful cause-and-effect linkage can be provided. 

As expected, a low cells/bead ratio caused a longer lag phase for the whole range of studied MC concentrations (3.12–6.34 g/L) and led to higher percentages of unoccupied MCs. Conversely, a higher cells/bead ratio led to a higher final cell density within the same culture duration. The findings align with data generated in previous studies [19,21,48,49]. For example, Souza et al. (2005) conducted a Design of Experiments (DoE) involving three parameters: agitation rate, cells/bead ratio, and FBS concentration. They established an empirical model wherein the final cell density showed a positive correlation with the cells/bead ratio parameter [21]. Lou et al., (2021) also demonstrated that raising the cells/bead ratio from 20 to 30 resulted in an increase in the final cell density from 1.8 × 10^6^ cells/mL to 2.3 × 10^6^ cells/mL and shortened the culture period by two days. However, elevating the ratio further to 50 cells/bead did not yield any significant changes in the attainable final cell count [49]. According to the developed models and digital twin, increasing the cells/bead ratio by more than 16 cells/bead has the potential to enhance the maximum achievable cell density. However, further experiments are necessary to confirm this theory, given the potential risk of cell aggregation associated with higher cells/bead ratios. Additionally, determining the maximum seeding density (cells/bead) requires striking a balance with a split ratio. Opting for a higher seeding density would require more bioreactors to reach the final large bioreactor for the scale of product manufacturing. Consequently, we refrained from adopting a higher cells/bead ratio in this study.

Theoretically, a higher concentration of MC has the potential to increase the available surface area for cell attachment, leading to a higher cell density at the same cells/bead ratio. However, our findings indicate an optimal MC concentration range (3.5–4.5 g/L) for maximum cell growth, which interestingly differs from the outcomes of some other previous studies [51,52]. Nevertheless, a study by Luo et al. (2021), who investigated the growth of Chinese perch brain cells (CPB) on Cytodex 1 [49], found a similar relationship. They explored an MC concentration range of 2 to 5 g/L and found no significant difference in achievable maximum cell concentration between 4 and 5 g/L of Cytodex 1 [49]. Moreover, higher MC concentrations were associated with increased occurrences of bead-to-bead collision and bead-to-reactor parts collision, posing a potential risk of damage to the cells [53,54]. Nienow et al. (2013) explained that the potential damage due to bead-to-bead collision and bead-to-impeller collision is proportional to MC concentration with the power of 1 and 2, respectively [55]. 

In MC-based cell culture, it is crucial to identify an optimal agitation speed that can facilitate MC suspension and homogenize culture in terms of substrate composition and temperature while avoiding harm to the cells [15,35]. As mentioned above, in our study, a final cell density of more than 2.0 × 10^6^ cells/mL was attained in the MC concentration range of 3.5–4.5 g/L, a seeding density of > 16 cells/bead, and an agitation speed of 90–100 rpm. Although lower agitation speeds may facilitate cell attachment to MCs, they can also result in MC clumping and sampling challenges [32]. Conversely, higher agitation speeds have the potential to cause cell damage. Previous research has indicated that freely suspended mammalian cells (sized 15–20 µm) are less sensitive to fluid dynamic stresses induced by agitation speed [35,56], whereas cells growing on MCs are more sensitive due to their attachment to relatively large particles that are prone to collisions with each other or with bioreactor parts, particularly the impeller [35]. This theory is corroborated by Nienow et al. (2014), who explained that the potential damage due to bead-to-bead collision or bead-to-impeller collision is proportional to agitation speed with power exponents of 4 and 4.5, respectively [55].

As noted in Section 2.10.1, cell death can occur when the average length of Kolmogorov microscale size (λ) is below two-thirds of the MC diameter. Conversely, if the λ is large enough, MCs will move in the direction of the eddy, which can help prevent shear damage [57]. As was shown in Table 1, the calculated λ across the agitation experiment range was more than two-thirds of λ. However, the observed decrease in cell growth at higher agitation speeds can be partially attributed to the damage resulting from the smallest Kolmogorov microscale (λ_min_) near the impeller [33]. This minimum microscale can be determined based on the maximum local specific energy dissipation rate, ε_Tmax_. There are two different equations for ε_Tmax_, which have been described in the literature [24]. The first formula uses a correlation with the diameter and height of the bioreactor, while the second formula correlates with the width of the impeller blade (Wi), which represents the volume swept out by the impeller during rotation.

(5) $$\varepsilon_{Tmax}=N_{P}\times \frac{N^{3}D_{i}^{2}{D_{T}}^{2}H}{V}$$

(6) $$\varepsilon_{Tmax}=N_{P}\times \frac{N^{3}D_{i}^{3}}{\frac{\pi }{4}\times W_{i}}$$

H = height at working volume (cm)
D_T_ = tank diameter (cm)
W_i_ = impeller blade width (cm).

The calculated value of λ_min_ (Equation (3)) with the ε_Tmax_ (Equations (5) and (6)) falls within the range of 62–72 µm for the maximum agitation speed of 130 rpm. The range is smaller than two-thirds of the minimum MC size, 90 µm. These findings provide a plausible explanation for the observed decrease in cell growth at higher agitation speeds.

### 4.5. Scale-Up Outlook

Our study demonstrates that employing optimized operational parameters during MC cultivation, coupled with in situ cell detachment, yields highly reproducible results in terms of growth, glucose consumption, and lactate production. The cells/bead ratio and MC concentration are identified as scale-independent parameters that can be controlled during scale-up. Regarding agitation speed, our findings suggest that optimized cell growth occurs at 1.125–1.25 × N_js_, making it a critical parameter for scaling up. This parameter can be empirically determined through direct sampling at various agitation speeds at each scale or through indirect methods, such as numerical techniques like computational fluid dynamics (CFD) simulations [58].

Based on our results and taking into consideration the capability of MA 104 cell line for at least 5 detachment–attachment cycles, we recommend scaling up in single-use bioreactors, starting with 3c Eppendorf bioreactors, followed by 10c and 50c, and eventually scaling to 250 L and 1000 L single-use bioreactors from ThermoFisher, as presented in Table 2. Beginning with a 3c bioreactor with 3 g/L of MC and achieving at least 2.0 × 10^6^ cells/mL at each scale while keeping CPPs in the determined design space, sequential scaling in different bioreactor sizes can be implemented. In this proposed scaling approach, each bioreactor, before seeding, has a 3 g/L MC concentration based on the final cultivation volume. However, after adding the seed from the previous bioreactor, the concentration approaches the concentration in the design space. 

To our knowledge, no preceding research has been conducted to establish a reliable protocol for scaling up MA 104 cell line on MCs using in situ cell detachment. Previous attempts by other groups to scale up MC-based cultures with in situ detachment have only demonstrated the capability of up to three cycles of detachment, with no evidence of the reproducibility of the data. Additionally, all data were reported for the Vero cell line instead of MA 104 cell line [14,17,24,26]. Furthermore, there are no reports on the achievement of multiple in situ detachments. Most studies did not perform cell growth optimization after detachment from MCs to determine the optimal conditions for a robust process. Wu and colleagues reported successful in situ detachment of Vero cells up to two times in a 200 L bioreactor, but the cell growth trend and final cell density achieved varied at each scale, and the final cell density was notably low, reaching only 0.7 × 10^6^ cells/mL at a 50 L bioreactor [17].

## 5. Conclusions

In conclusion, our study unveils effective strategies for MC cultivation of MA 104 cell line, crucial for scalable vaccine manufacturing. The consecutive cell detachment–attachment method emerged as a robust approach, showcasing the cells’ ability to undergo multiple in situ detachments and efficiently recolonize Cytodex 1. This method holds promise for seed training and cell propagation, providing a viable path for attaining sufficient cells for antigen manufacturing. Our research emphasizes the need for optimized cell detachment methods and explores novel aspects such as repetitive recolonization of spent MCs, showcasing MA 104 cells’ ability to resettle on the spent Cytodex 1 without additional cleansing steps.

The DoE study and hybrid process model reveal critical parameters influencing cell densities, offering insights for achieving optimal growth that can be used as control sheets to estimate outcomes under unfavorable conditions, for example, lower seeding densities or different microcarrier concentrations. 

The proposed scaling-up strategy using single-use bioreactors, initiated with smaller volumes and progressing gradually to larger scales, provides a practical guide for researchers and industry practitioners aiming to implement efficient and reproducible MC-based cell culture systems. Overall, our findings contribute valuable knowledge to the field, offering a comprehensive understanding of MA 104 cell behavior and providing practical recommendations for successful and scalable MC-based cell cultivation.

## Figures and Tables

**Table 2:** Proposed scaling-up approach for microcarrier-based cell culture in single use bioreactor.

	3c	10c	50c	250 L	1000 L
Bioreactor WV * (L)	1.25–3.75	3.3–10	18–40 L	125–250	500–1000
Actual WV ** (L)	2.5	10	40	190	900
Volume taken from previous culture (L)	- ***	2.5	10.0	40.0	190.0
Actual MC Conc. (g/L)	3.0	3.8	3.9	3.8	3.8
Actual cells/bead	20.0	21.7	20.7	17.9	18.0

* Working volume; ** designed working volume; *** seed from static culture; seed transferring criteria: total cell density achievable at each step: 2.0 × 10^6^ cells/mL; total cell recovery after in situ cell detachment: 70%; defined seeding cells/bead ratio: 20 cells/bead. MC concentration in each prepared bioreactor before seeding: 3 g/L.

## Data Availability

Data are contained within the article and Supplementary Materials.

## Supplementary Materials

The following supporting information can be downloaded at: https://www.mdpi.com/article/10.3390/bioengineering11030268/s1, Figure S1: Cytodex 1 diameter distribution; Figure S2: Microscopic observation of cell growth on MCs; Figure S3: Percentage of free suspended cells during the first 6 h of culture in the spinner; Figure S4. (a) Simulated result of cell growth during the culture for MC range of 3.5–4.5 g/L at seeding density and agitation speed of 16.5 cells/bead and 95 rpm, respectively. (b) Simulated result of cell growth during the culture for an agitation speed range of 90–100 rpm at seeding density and MC concentration of 16.5 cells/bead and 4 g/L, respectively (*p*-value < 0.05); Figure S5: Impact of removing FBS in the second medium exchange (ME) on cell growth after in situ cell detachment; Table S1: Conducted cultivation runs of the designed experiments, File S1.xlsx.
