# Supplementary material for: Seed Train Optimization in Microcarrier-Based Cell Culture Post In Situ Cell Detachment through Scale-Down Hybrid Modeling
Source: Bioengineering (Basel). 2024 Mar 9;11(3):268. doi: 10.3390/bioengineering11030268 (PMC10968011; doi:10.3390/bioengineering11030268)
Supplement: Supplementary file 1 [file bioengineering-11-00268-s001.zip › bioengineering-2858591-supplementary.pdf]

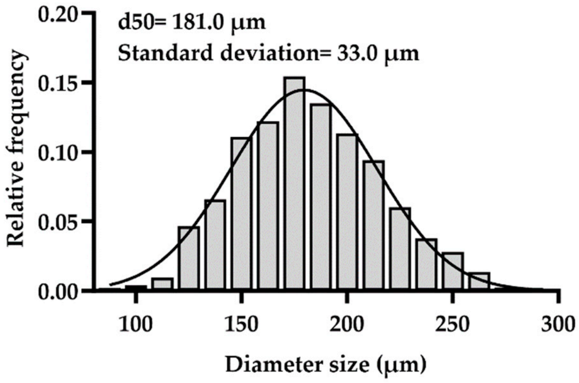

Supplementary Figure S1. Cytodex 1 diameter distribution.

a)

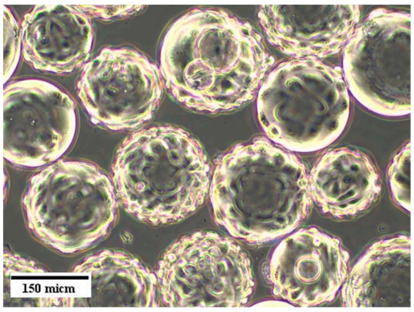

b)

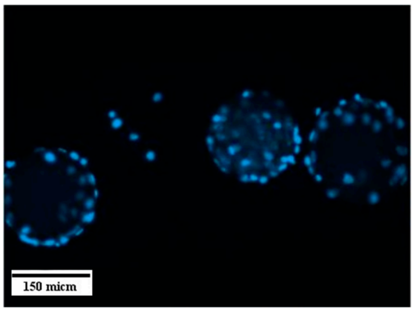

Supplementary Figure S2. Microscopic observation of cell growth on microcarrier after multiple in situ cell detachment (at 5th day of culture after in situ cell detachment): a) optical microscope observation of the cells on microcarrier and b) fluorescence images (Zeiss—Axio Observer Z1, Germany) of live cell staining of the MA 104 cells with Hoechst 33342 (Thermo Fisher, Massachusetts, USA).

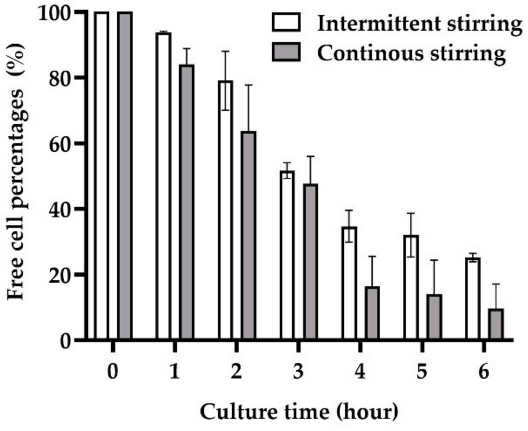

Supplementary Figure S3. Percentage of free suspended cells during the first 6 hours of culture in the spinner. Two stirring conditions: continuous (60 rpm) and intermittent (15 min on (60 rpm), 30 s off) and with an initial cell density of 200,000 cells/mL (15 cells/bead).

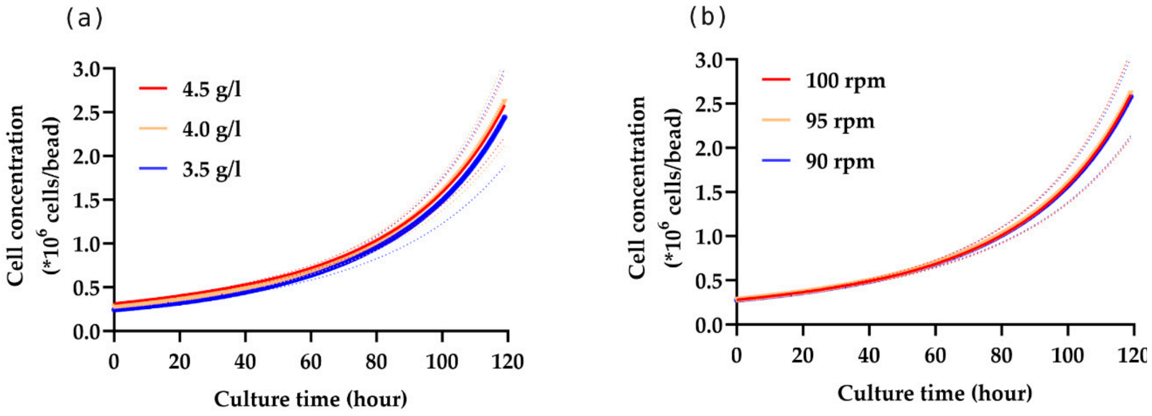

Supplementary Figure S4. (a) Simulated result of cell growth during the culture for MC range of 3.5-4.5 g/l at seeding density and agitation speed of 16.5 cells/bead and 95 rpm, respectively. (b) Simulated result of cell growth during the culture for an agitation speed range of 90-100 rpm at seeding density and MC concentration of 16.5 cells/bead and 4 g/l, respectively (p-value < 0.05).

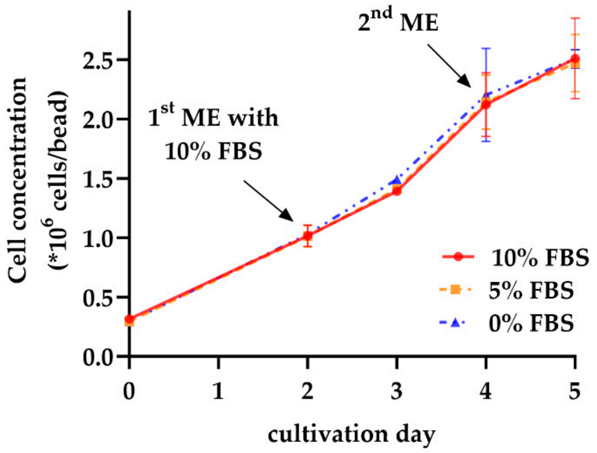

Supplementary Figure S5. Impact of removing FBS in the second medium exchange (ME) on cell growth after in situ cell detachment.

Supplementary Table S1. Conducted cultivation runs of the designed experiments.

Run No.	MC Conc. (g/l)	Seeding density (cells/bead)	Agitation speed (rpm)	Actual MC Con. (g/L)	Actual seeding density (cells/bead)
**Run 1**	3	20	70	3.8	15.77
**Run 2**	3	4	130	3.26	3.68
**Run 3**	5	20	130	6.34	15.77
**Run 4**	5	4	70	5.35	3.74
**Run 5**	3	4	70	3.12	3.85
**Run 6**	4	12	100	4.47	10.74
**Run 7**	4	20	100	4.78	16.72
**Run 8**	5	4	130	5.2	3.85
**Run 9**	4	4	70	4.25	3.76
**Run 10**	4	4	100	4.25	3.76
**Run 11**	4	20	100	5.16	16.02
**Run 12**	5	12	100	5.16	16.02
**Run 13**	3	12	100	3.56	10.44
**Run 14**	4	12	70	4.75	10.44
**Run 15**	5	12	130	5.96	10.07
**Run 16**	4	12	130	4.77	10.07
**Run 17**	4	12	130	4.77	10.07
**Run 18**	3	12	100	3.58	10.07
**Run 19**	4	12	100	4.84	9.92
**Run 20**	3	20	130	3.98	15.08
**Run 21**	5	12	70	6.1	10.7
